# Understanding the Role of Fibrotic Scarring in Shaping the Lesion Site and Neural Repair After Spinal Cord Injury

**DOI:** 10.3390/cells15131135

**Published:** 2026-06-23

**Authors:** Camilo Jubino Londoño, Binhai Zheng

**Affiliations:** 1 Neurosciences Graduate Program, University of California San Diego, La Jolla, San Diego, CA 92093, USA; clondono@health.ucsd.edu; 2 Department of Neurosciences, School of Medicine, University of California San Diego, La Jolla, San Diego, CA 92093, USA; 3 VA San Diego Research Service, San Diego, CA 92161, USA

**Keywords:** fibrotic scar, spinal cord injury, neural repair, lesion site, fibroblast, fibrosis

## Abstract

Following spinal cord injury (SCI), a complex lesion scar forms at the injury site that matures and remodels over weeks, profoundly influencing neural repair and functional recovery. This lesion consists of a fibrotic scar at its core surrounded by an astrocytic scar (or border). While the astrocytic scar has been extensively studied for decades, the fibrotic scar has only recently emerged as a critical player in post-injury pathophysiology. Fibrotic scarring plays a dual role: it contributes to tissue stabilization and limits secondary damage, yet its persistence can pose a barrier that inhibits axonal regeneration and hinders recovery. Despite growing interest, key aspects of fibrotic scar formation and function remain poorly understood. This review synthesizes the current knowledge of fibrotic scarring after SCI, including its temporal progression, cellular composition, molecular mechanisms, and interactions with other cell types at the injury site, and we discuss emerging therapeutic strategies targeting fibrosis. We further highlight critical knowledge gaps and outline future directions to define how fibrotic scarring shapes the injury microenvironment and influences neural repair.

## 1. Introduction

Fibroblasts are critical regulators of tissue homeostasis, continuously surveying biomechanical properties and maintaining extracellular matrix (ECM) integrity to preserve normal tissue architecture and function [1,2]. Following severe tissue injury, these homeostatic mechanisms are disrupted, prompting fibroblasts to initiate a coordinated wound-healing response [1,2]. This involves fibroblast activation, migration, proliferation, and extensive ECM remodeling, including the production of ECM proteins that serve as a structural scaffold to restore tissue integrity as closely as possible to its pre-injury state [1,2].

In the context of SCI, this orchestrated wound healing becomes dysregulated, leading to fibrosis. Fibrosis is characterized by the persistent presence and activation of fibroblasts, coupled with excessive and abnormal deposition of ECM proteins, resulting in tissue properties that markedly differ from the healthy spinal cord [3,4,5,6,7]. This aberrant response culminates in the formation of a chronic fibrotic scar, a defining feature of the long-term SCI lesion site [5,8,9,10]. Although fibrotic scarring has long been associated with impaired axonal regeneration and poor functional recovery after SCI, research efforts have historically focused predominantly on the astrocytic scar. Only recently has a greater appreciation for the role of fibroblasts in central nervous system (CNS) injury revealed significant knowledge gaps regarding the cellular origins, molecular mechanisms, and intercellular interactions that govern fibrotic scar formation and maintenance [5]. A comprehensive understanding of these processes is essential for developing effective therapies capable of modulating fibrotic scarring while preserving its beneficial functions. In this review, we discuss the identity and origins of fibrotic-scar-forming cells, the temporal dynamics of scar formation, fibrotic scar intercellular crosstalk, and valuable insights from scar-free or minimally scarring models in regenerative systems. By integrating these perspectives, we aim to illuminate key mechanisms and identify promising directions for future research in SCI.

## 2. The Cellular Identity of Fibrotic-Scar-Forming Cells

The identity of cells that form the SCI fibrotic scar has been the subject of long-standing discussion. Early studies using penetrative brain injury models proposed that the fibrotic scar originated from infiltrating meningeal cells [11,12]. However, robust fibrotic scarring in non-penetrative SCI, where the dura and meninges remain intact, challenged this view and raised questions about whether other cell types contribute [13]. Fibroblasts and pericytes have emerged as primary candidates involved in SCI fibrotic scar formation. Despite differences in location, morphology, and physiological functions, their transcriptional profiles substantially overlap, making them difficult to reliably distinguish [14,15]. This is compounded by technical limitations in isolating pure perivascular populations without cross-contamination. Single-cell RNA sequencing (scRNA-seq), lineage-tracing, and improved cell isolation have sharpened our understanding of the contribution of fibroblasts and pericytes in fibrotic scar formation after SCI [16,17,18].

### 2.1. Genetic Markers of Fibroblasts and Pericytes

Fibroblasts are mesenchymal cells that survey and remodel the ECM to provide structural support to tissues [1,2,19]. Fibroblast-specific markers have generally reflected their central role in ECM production, homeostasis, and remodeling, such as collagen type I alpha 2 chain (Col1a2). Platelet-derived growth factor receptor alpha (PDGFRα) has also emerged as a robust fibroblast-enriched marker [20,21]. A comprehensive scRNA-seq study of vascular and vessel-associated cells from adult mouse brains applied PDGFRα as a working fibroblast marker and identified fibroblast-associated expression of ECM-associated proteoglycan and regulator genes, such as Decorin, lumican, and Lama1 [17]. ScRNA-seq of PDGFRβ^+^/GLAST^+^ perivascular cells isolated from uninjured spinal cords revealed a fibroblast population also showing strong enrichment for Pi16, Periostin, and Fibronectin 1 [18]. Similarly, scRNA-seq of PDGFRβ+ cells isolated after SCI identified a fibroblast-specific gene signature characterized by elevated expression of Mmp2, Matn2, Cyp1b1, and Clmp [16]. Together, these datasets reinforce a core set of fibroblast-enriched transcripts, although further validation across SCI models and time points is needed to determine their stability and specificity in the evolving fibrotic scar.

Pericytes are mural cells that ensheathe the endothelium of CNS blood microvessels, providing critical support for blood–brain barrier integrity, vascular stability, and regulation of cerebral blood flow [22,23]. Pericyte markers have largely emerged from studies of blood–brain barrier development and maintenance. Classic candidates identified through immunofluorescence and transgenic models include CSPG4 (NG2) [24,25], desmin [26,27], Anpep (CD13) [28,29], RGS5 [30,31], Dlk1 [32], and Cd248 [33]. Subsequent microarray and scRNA-seq studies have additionally identified Atp13a5 [34,35], Abcc9 [32,34], and Kcnj8 [32,34] as pericyte-enriched markers. Since then, scRNA-seq, lineage-tracing with transgenic mouse models, and improved purification strategies have continued to validate and confirm pericyte markers [17,18,35,36,37,38,39]. In the context of SCI, perivascular cells isolated from PDGFRβ^+^/NG2^+^ mice validated vitronectin (Vtn) as a pericyte-specific gene [36], a finding supported by subsequent scRNA-seq datasets [17,18,35,37]. Slc1a3 (encoding GLAST), initially recognized for its expression in astrocytes, retinal Müller glia, and hippocampal neural stem/progenitor cells, was also found to label a subset of pericytes in the spinal cord [40,41]. PDGFRβ^+^/GLAST^+^ perivascular cells isolated and sequenced from uninjured spinal cords demonstrated pericyte-enriched markers of Vtn, Anpep, Atp13a5, Kcnj8, Rgs5, and CSPG4 at higher levels relative to fibroblast clusters [18].

Notably, several markers are expressed by both populations, limiting their specificity. PDGFRβ, initially associated with pericytes through genetic deletion studies, is now recognized to be broadly expressed across multiple perivascular mesenchymal cells, including fibroblasts, thereby reducing its utility as a standalone discriminator [42,43,44]. Anpep, or CD13, was first identified exclusively in pericytes by microsequencing and RT-PCR of cerebral microvessels [29]. However, differential expression analysis to construct fibroblast and mural cell-specific markers did not include Anpep as a mural cell-specific marker, suggesting its expression in fibroblast cells [15]. Recently, Slc1a3 (GLAST) was also shown to be expressed in fibroblasts [17,18]. Future studies employing lineage tracing, high-resolution spatial transcriptomics, and functional validation will be critical to resolve the remaining ambiguities in marker specificity. Markers for fibroblasts and pericytes are summarized in Table 1.

### 2.2. Pericytes in Fibrotic Scarring

A breakthrough in the contribution of pericytes in fibrotic scarring was the identification of a subset of pericytes shown to localize to the fibrotic scar after SCI [40]. Using a GLAST-driven CreER line, recombined GLAST^+^ cells detached from the vascular wall as early as 3 days post dorsal hemisection SCI, migrated into the lesion core, extended thin processes, and persisted chronically [40]. These cells were termed as pericytes due to their association with parenchymal blood vessels, encasement within the vascular basal lamina, ultrastructural features typical of pericytes, and expression of PDGFRα, PDGFRβ, and CD13/Anpep [40]. Because only ~10% of all spinal cord pericytes recombined in the GLAST-CreER model, this subpopulation was designated as “type A pericytes” [40]. Interestingly, after SCI, a majority of GLAST^+^ cells do not express the pericyte marker NG2, suggesting that GLAST^+^/NG2^−^ cells play a role in SCI fibrotic scarring [53]. However, fibroblasts also express CD13/Anpep, PDGFRα, PDGFRβ, and GLAST, while not expressing NG2, raising questions about whether type A pericytes are fibroblasts [18]. Regardless, subsequent studies have validated the contribution of GLAST^+^ cells to fibrotic scarring after SCI [18] as well as in traumatic brain injury, multiple sclerosis, and stroke [53]. Interestingly, lineage tracing of vascular smooth muscle cells (αSMA^+^) and pericytes (NG2^+^) in an EAE model of multiple sclerosis (MS) shows minimal signal within lesion areas [48,54]. The precise contribution of pericytes and their transition into activated, scar-forming cells after SCI remains incompletely resolved and warrants further investigation.

ScRNA-seq data have also provided evidence for the involvement of pericytes in fibrotic scar formation after SCI. For example, scRNA-seq of GLAST^+^/PDGFRβ^+^ cells at 3 and 5 dpi identified distinct pericyte and activated pericyte clusters expressing canonical pericyte markers such as Atp13a5, Kcnj8, and Cspg4 [18]. Interestingly, pseudotime trajectory analysis shows a shift in pericyte identity toward a fibroblast-like state, with progressive downregulation of pericyte-associated genes (Atp13a5, Cspg4) and upregulation of fibroblast markers (Col1a1, Col8a1, Fn1) [18]. ScRNA-seq of PDGFRβ^+^ cells after SCI revealed a pericyte cluster enriched for Abcc9 and Rgs5 [16], while thoracic contusion datasets showed a pericyte population marked by Kcnj8 expression [55]. Spatial transcriptomics following right lateral hemisection further supported the presence of pericyte-signature cells, specifically implicating type A pericytes in scar formation [56]. Finally, snRNA-seq analyses have identified a distinct pericyte cluster in the injured spinal cord [57]. Pericyte genetic labeling SCI studies are summarized in Table 2.

### 2.3. Fibroblasts in Fibrotic Scarring

Fibroblasts have long been recognized for their central role in ECM production and deposition. In the uninjured spinal cord, perivascular fibroblasts are reliably labeled using Col1a1-GFP mice and are predominantly located on large-diameter blood vessels, where they colocalize with PDGFRβ and CD13 [13]. Following SCI, fibroblasts rapidly migrate into the injury site by 4 days post-injury (dpi) and exhibit complete colocalization with PDGFRβ [13]. To distinguish these cells from pericytes, which also express PDGFRβ and produce ECM proteins, a dual-reporter model (Col1a1-GFP; NG2CreER^T2^; tdTomato) was employed. In this system, Col1a1^+^ cells localized primarily to large vessels and were negative for the pericyte marker NG2, whereas NG2^+^ cells were restricted to small vessels [13]. After SCI, Col1a1^+^ cells accumulated at the injury site and did not colocalize with NG2^+^ cells [13]. In fact, NG2^+^ cells were primarily located outside of the injury site [13]. Recent lineage-tracing studies with Col1a1^+^ promoters have confirmed the presence of these cells in the fibrotic scar following contusive and complete crush SCI [18]. Future studies will need to clarify the relative contributions of pre-existing versus injury-induced fibroblast populations and the relative contribution of meningeal and perivascular fibroblasts to SCI fibrotic scarring.

ScRNA-seq of GLAST^+^/PDGFRβ^+^ cells at 3 and 5 dpi identified clear fibroblast clusters, including activated fibroblasts and myofibroblasts, supported by strong enrichment of Pi16 and multiple ECM-associated genes [18]. Similarly, scRNA-seq of PDGFRβ^+^ cells after SCI revealed fibroblast and dividing fibroblast clusters, characterized by expression of Pi16, PDGFRα, and Lumican in the former, and proliferation markers such as Cdk1, Pclaf, and Mki67 in the latter [16]. Notably, at both 5 and 14 dpi, the proportion of fibroblasts was substantially higher than that of pericytes or vascular smooth muscle cells [16]. ScRNA-seq after thoracic contusion injury also identified a fibroblast cluster marked by strong Col1a1 expression and showed unique interactions with macrophages through IL1α/β, Vegfa/b, PDGFA, and TGFβ1 signaling [55]. Finally, spatial transcriptomics following right lateral hemisection injury revealed a fibroblast-like cluster with prominent expression of Col1a1 and Col1a2 [56]. Fibroblast genetic labeling SCI studies are summarized in Table 2.

## 3. Signaling Pathways Regulating Fibroblast Activation

Fibroblasts are primarily located in the perivascular space, meninges, and dura mater of the uninjured spinal cord. Following tissue injury, however, fibroblasts shift from a quiescent, surveillance state to an activated, extracellular matrix-producing phenotype [5,59,60]. This cell state transition is tightly regulated by a set of signaling pathways. A detailed understanding of these pathways is essential for developing targeted strategies to modulate fibroblast activation, control the extent of fibrotic scarring, and limit the chronic persistence of scar-forming cells after SCI.

### 3.1. TGF-β

Transforming growth factor-beta (TGF-β) signaling is one of the most well-characterized mediators of fibroblast activation and myofibroblast differentiation following tissue injury [60,61,62,63]. In peripheral organs such as the lung, kidney, and skin, TGF-β is widely recognized as a central driver of pathological fibrosis, and accumulating evidence suggests this role extends to the CNS [60,61,62,63,64]. For example, TGF-β mRNA is rapidly induced at the injury site in multiple CNS injury models, including traumatic brain injury, cerebral ischemia, and SCI [64,65,66,67,68]. Recent spatial and single-cell transcriptomic studies have provided higher-resolution insights. In a photothrombotic (PT) stroke model, spatial sequencing revealed that Col1a1-enriched fibroblast clusters at 7 dpi exhibited elevated TGF-β signaling and proliferation scores compared to fibroblasts at 21 dpi [69]. SnRNA-seq of the cortex after PT stroke identified TGFB1 as the dominant ligand likely driving myofibroblast transition, with disease-associated microglia and scar-associated macrophages emerging as the primary cellular sources [69]. These results demonstrate the role of TGF-β signaling in activating fibroblasts and promoting fibrotic scar formation after stroke.

ScRNA-seq of PDGFRβ^+^ cells from SCI tissue demonstrated significant enrichment of TGF-β signaling pathways in fibroblasts at both 5 and 14 dpi, as revealed by KEGG pathway analysis [16]. Importantly, ligand–receptor interaction scores for TGF-β were substantially higher in fibroblasts than between pericytes/vascular smooth muscle cells, highlighting a fibroblast-specific role for TGF-β signaling in fibrotic scar formation [16]. ScRNA-seq of cells isolated from GLAST^+^/PDGFRβ^+^ injured spinal cords showed that both activated fibroblasts and myofibroblasts share strong gene ontology enrichment for TGF-β signaling pathways [18].

### 3.2. PDGF

Platelet-derived growth factor (PDGF) signaling is another known mediator of tissue repair and fibrosis [5,70,71]. Similar to TGF-β, PDGF signals can act on its receptors to activate mesenchymal cells, such as fibroblasts and pericytes. In fact, PDGF signaling has been associated with fibrosis of the kidney, liver, skin, intestine, heart, eye, and lungs, similar to TGF-β signaling [70]. For example, PDGFRα activation using knock-in mice resulted in spontaneous fibrosis in skeletal muscle, heart, lung, kidney, and intestine tissues [72]. Importantly, PDGF signaling has been implicated in fibrosis in the CNS. Cells expressing PDGF receptors alpha and beta (PDGFRα, PDGFRβ) populate the injury site in TBI and SCI [13,16,18,40,73]. ScRNA-seq of PDGFRβ^+^ cells after SCI revealed elevated PDGF signaling among pericytes, endothelial cells, microglia, and macrophages at 5 and 14 dpi [16]. Future experiments perturbing PDGF signaling in SCI models will provide functional evidence of its role in fibrotic scarring.

### 3.3. CXCL4

In addition to TGF-β and PDGF, inflammatory chemokines regulate fibroblast recruitment, activation, and fibrotic scar formation. Prior literature has implicated CXCL4 signaling in fibrotic disorders by driving the transition of cells into myofibroblasts [74]. In the context of SCI, in vitro and in vivo data suggest that macrophage-derived CXCL4 promotes pericyte-to-myofibroblast transition [75]. In vitro treatment of pericytes with CXCL4 upregulates myofibroblast markers Acta2 and Col1a1, while downregulating pericyte markers PDGFRβ and NG2 [75]. In vivo, intrathecal injection of neutralizing anti-CXCL4 antibodies after SCI suppresses the upregulation of α-SMA, collagen I, laminin, and fibronectin, resulting in an attenuated fibrotic scar [75].

### 3.4. Other Signaling Pathways

In addition to TGF-β, PDGF, and CXCL4 signaling, several other pathways have been implicated in fibrotic scarring after SCI, including Eph/ephrin signaling, integrin β1, N-cadherin, and PlexinB1/B2 [76,77,78,79,80,81,82]. Ephrin-B2/EphB2, for example, contributes to the formation and maintenance of the sharp boundary between the astrocytic and fibrotic scars [79,80,82]. Similarly, integrin β1 and N-cadherin mediate astrocyte–fibroblast adhesion in response to fibrotic ECM, while PlexinB1/B2 signaling supports wound corralling and injury border organization [76,77,82]. However, the precise mechanisms by which these pathways may modulate fibroblast activation, proliferation, migration, or ECM production in the context of SCI remain incompletely elucidated and warrant further investigation.

## 4. Fibrotic Scarring Dynamics in SCI

The SCI fibrotic scar structure is the culmination of dynamic, orchestrated injury site remodeling. This involves the coordinated activity of multiple cell types, including the influx of inflammatory and immune cells, the recruitment and activation of perivascular and meningeal cells, and the progressive accumulation and organization of ECM proteins produced within the lesion site. Here, we review the key cellular events that occur during the acute, maturing, and chronic phases of fibrotic scar formation after SCI.

### 4.1. Acute Phase (Day 0–7)

The acute phase is defined as the first week following the primary mechanical injury. Relative to the injured cord, the uninjured spinal cord significantly lacks ECM proteins and meningeal-derived cells within the white and grey matter parenchyma [5,9,13,48]. In the first few days after SCI, there is extensive cell death, cellular debris, immune cell infiltration [56,83], and absence of blood vessels, fibroblasts, and pericytes at the injury site [13,40,53]. Both fibroblasts and pericytes remain tightly associated with the vascular wall during the first two days, indicating they require specific recruitment signals to localize at the injury site [13,40]. At 3 dpi, blood vessels begin to sprout, and type A pericytes and fibroblasts begin to appear within the injury site [13,40,56]. During this period, fibronectin levels increase modestly and diffusely throughout the injured spinal cord [84]. By 5 dpi, type A pericytes detach from the vessel wall, express proliferative markers, and upregulate myofibroblast markers [40,53], a behavior conserved in both penetrative and non-penetrative SCI models. PDGFRβ^+^ cells also appear within the injury site by 5 dpi in both complete crush and transection models, where they express the myofibroblast marker Acta2, consistent with an activated and proliferative state [16].

Bulk-RNA seq of SCI tissue at acute timepoints reveals a sudden decrease in neuronal-related gene modules and an increase in immune and gliogenesis modules after SCI [83]. CD45, a pan-leukocyte marker, sharply increases after SCI and plateaus after the acute phase, while markers for microglia exhibit steady increases during the acute phase, illustrating the sudden immune response after SCI [83]. Further studies are needed to clarify the specific signals produced by early immune cell influx (0–3 dpi) that recruit and activate fibroblasts and type A pericytes.

### 4.2. Maturing Phase (Day 7–14)

During the maturing phase, fibroblasts, type A pericytes, and ECM proteins within the injury site undergo robust expansion and organization. Type A pericytes reach their peak abundance around 9 dpi, coinciding with the progressive structuring and contraction of the astrocytic scar, which corrals cells within the lesion site [40]. Similarly, PDGFRβ^+^ cells significantly increase in number between 5 and 14 dpi in both complete transection and crush SCI models, with notably higher numbers observed in the transection model [16]. In contusive SCI, fibroblasts become clearly confined within the lesion core by the surrounding GFAP^+^ astrocytes by 7 dpi [13], along with fibronectin deposition shifting from diffuse to condensed and fibrillar, indicating active matrix assembly [84]. At this stage, a distinct fibrotic compartmentalization emerges, consisting of a fibrotic scar populated by fibroblasts and ECM proteins, sharply demarcated by the forming astrocytic border [13,56]. The emerging segregation between the astrocytic border and fibrotic scar is thought to involve the formation of a basal lamina [85,86]. This structure is prominently observed surrounding GLAST^+^ cells in complete crush models [53], as well as PDGFRβ^+^ cells [16]. Both cell populations show intimate spatial association with key ECM components, including fibronectin and collagen I, highlighting their active role in matrix remodeling during this critical maturation window.

### 4.3. Chronic Phase (Day 14-Onward)

**Figure 1:**
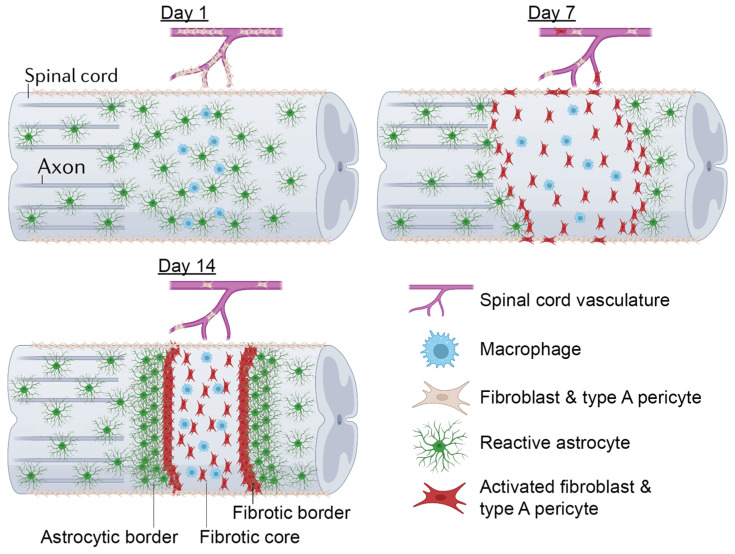
**Temporal progression of fibrotic scar formation after SCI.** Schematic illustrating the dynamic cellular changes underlying scar formation following SCI. This image was created with BioRender.com.

In the chronic phase of fibrotic scar formation following SCI, which begins around 14 dpi and persists chronically, the scar structure largely stabilizes with minimal further active remodeling or expansion compared to the maturing phase. The fibrotic scar remains sharply compartmentalized from the surrounding astrocytic border. In contusive SCI, Col1a1^+^ perivascular fibroblasts remain largely stable in number, distribution, and morphology beyond 14 dpi, with no significant further shrinkage of the injury site or alterations in scar architecture [13]. The fibrotic scar continues to consist of distributed Col1a1^+^ and PDGFRβ^+^ cells embedded within condensed, fibrillar ECM proteins [13,16]. Fibronectin matrix assembly also remains consistent with the 14 dpi state, characterized by dense, fibrillar deposition without notable further accumulation or reorganization [84]. Interestingly, long-term assessment up to 7 months post-injury reveals a gradual decline in type A pericytes (approximately 50% reduction from 14 dpi), accompanied by modest continued shrinkage of the injury site in dorsal hemisection models, but not in contusion injuries [40]. Regardless, the overall injury site structure persists, with fibroblasts/pericytes confined to the injury site and surrounded by the astrocytic border [40]. The bidirectional crosstalk between fibroblasts and astrocytes, particularly the signals that maintain the sharp astrocytic–fibrotic border, is still not fully elucidated. The progression of fibrotic scar formation is summarized in Figure 1.

## 5. Multicellular Interactions at the SCI Lesion Site

The injury site after SCI is a complex, dynamic microenvironment shaped by the intricate interplay among multiple cell types [9,87,88]. Many of these cells maintain direct or indirect relationships with fibroblasts and the developing fibrotic scar [10]. A deeper understanding of how astrocytes, macrophages, and microglia interact with fibroblasts is therefore essential, as these interactions critically influence the injury site’s capacity to support or restrict axon repair and regeneration.

### 5.1. Fibroblast Interactions with Immune Cells

SCI causes a rapid influx of immune cells to the lesion site, where they contribute to debris clearance, cytokine and chemokine signaling, and the recruitment of additional cells, including those involved in fibrotic scar formation [89,90]. Emerging evidence reveals intimate temporal and spatial crosstalk between immune cells and fibroblasts that actively drives fibrotic scar development. Temporal dynamics after SCI show sparse pericyte and fibroblast presence and modest immune cell presence at 3 dpi [58,91]. Both populations increase substantially by 7 dpi and mature into a structured fibrotic scar by 14 dpi [58,91]. At 14 dpi, CD11b^+^ immune cells, Mac2^+^ macrophages, and CD68^+^ activated macrophages associate closely with fibroblasts [16,58,91]. To distinguish the relationship of fibroblasts with resident microglia and hematogenous macrophages, bone marrow chimeric models allow for differential labeling of hematogenous macrophages and microglia [58]. By 14 dpi, the fibrotic scar core is predominantly populated by hematogenous macrophages, whereas microglia remain largely restricted to the peripheral edges of the astrocytic scar [58]. This spatial segregation demonstrates that infiltrating macrophages, rather than resident microglia, are most closely associated with the fibrotic core both temporally and spatially. Bulk RNA-seq comparing uninjured and 3 dpi spinal cord tissue revealed significant upregulation of Cxcl4 expression post-injury, which was found to be predominantly expressed by Spp1^+^ Fn1^+^ profibrotic macrophages [91]. This early macrophage-derived CXCL4 likely promotes pericyte recruitment and subsequent myofibroblast transition, thereby reinforcing fibrotic scar formation [91]. Further investigation is required to fully determine how bidirectional signaling between specific macrophage subpopulations and fibroblasts evolves from the acute to chronic phases of fibrotic scar formation.

### 5.2. Fibroblast Interactions with Astrocytes

The astrocytic scar and fibrotic scar represent the two primary scarring phenomena after SCI. Their proximity critically shapes scar architecture, barrier function, and the overall permissiveness of the lesion microenvironment for neural repair [9,10,82,92]. Initial evidence of astrocyte–fibroblast relationships came from their distinct organization in the mature scar. By 14 dpi, the astrocytic scar lies immediately adjacent to a dense rim of Col1a1^+^ fibroblasts, forming a sharp boundary that appears to restrict serotonin-positive axon growth after contusive SCI [13]. GFAP^+^ astrocytes of the astrocytic scar express the ephrin-B2 ligand, while fibronectin^+^ fibrotic cells of the fibrotic scar express its receptor EphB2 [78]. Ephrin-B2/EphB2 signaling has been suggested to mediate molecular crosstalk between astrocytes and fibroblasts [78,79,80,81].

Further nuance arises from differences among astrocyte subpopulations. Reactive astrocytes predominantly associate with collagen I-negative regions, whereas scar-forming astrocytes show strong colocalization with collagen I [82]. This suggests fibroblast-derived collagen I contributes to the conversion of reactive astrocytes into scar-forming astrocytes, potentially through integrin-mediated ECM sensing, thereby reinforcing the astrocytic border. Integrin β1 antibody treatment reduced astrocyte scar formation, improved functional recovery, and increased the number of 5-HT, GAP43, and tyrosine hydroxylase-positive axonal fibers both within and beyond the injury site [82]. Similarly, antibody blockade of N-cadherin attenuated astrocyte scar formation to a comparable extent, supporting the role of this pathway in astrocytic responses to fibrotic ECM [82].

Plexin signaling has recently provided evidence for the crosstalk between astrocytes, macrophages, and fibroblasts [76,77]. PlexinB2 conditional knockout in injury-activated microglia and macrophages significantly increased lesion site volume, impaired wound corralling, and disrupted astroglial–fibrotic border formation [77]. Similarly, PlexinB1 conditional knockout in astrocytes disrupted border formation and increased fibroblast activity [76].

## 6. Past Interventions Targeting Fibrotic Scarring After SCI

### 6.1. Pharmacological Approaches

Pharmacological strategies have been employed to modulate fibrotic scarring after SCI by targeting key signaling pathways involved in fibroblast activation and ECM deposition. Many early studies have directly targeted the collagenous basement membrane that forms after SCI, with mostly positive results in axonal regeneration and behavioural recovery [93,94,95,96,97,98]. Knockout of periostin, an ECM protein, exhibited decreased PDGFRβ expression, reduced pericyte proliferation, improved Basso Mouse Scale (BMS) scores, and increased axons crossing the lesion site after contusive SCI [99]. An antagonistic monoclonal antibody against periostin resulted in improved BMS locomotor recovery and significantly smaller fibrotic scars [99]. However, the effects were time-dependent; when antibody administration was delayed until after 2 weeks post-injury, no improvement in functional recovery was observed, underscoring a critical therapeutic window during the acute and subacute phase of scar formation [99]. The optimal therapeutic window and level of fibrotic scar modulation needed to promote regeneration without compromising tissue integrity must be considered in future studies.

More recently, neutralization of TGF-β with a specific antibody significantly increased the density of 5-HT and βIII-tubulin-positive axons crossing the injury site, enhanced glial scarring, reduced ECM markers, and improved functional recovery [100]. This treatment also lowered both active and total TGF-β1 levels in serum and spinal cord tissue, while decreasing the number of pSmad2^+^ PDGFRβ^+^ pericytes, confirming effective suppression of TGF-β-driven fibroblast activation [100]. Pharmacological inhibition of PDGFRβ signaling has also proven effective in attenuating fibrotic scar formation. Intrathecal delivery of the PDGFRβ inhibitor SU16f reduced PDGFRβ expression, fibronectin and laminin deposition, lesion size, and proliferation of PDGFRβ^+^ cells at 28 dpi [101]. Additionally, SU16f treatment disrupted the astroglial–fibrotic border, resulting in a more disordered interface [101]. Daily intrathecal injections of imatinib, an inhibitor of PDGF signaling, promoted locomotor recovery, increased the number of axons crossing the lesion site, preserved NeuN^+^ neurons, and reduced PDGFRβ expression, ECM markers, and FSP1^+^ fibroblasts [102].

### 6.2. Genetic Approaches

Genetic approaches have enabled precise, cell-specific, and gene-specific manipulation of scar-forming cells, offering rigorous experimental control and deeper mechanistic insights into the contributions and functional importance of fibrotic scarring after SCI. One strategy has targeted the astrocyte-fibroblast interface, such as through Eph/ephrin signaling. Astrocyte-specific deletion of ephrinB2 in a lateral hemisection model allowed corticospinal tract axons to grow closer to the lesion center, although they did not cross the fibrotic core [79]. These mice exhibited a smaller astroglial scar and faster BMS locomotor recovery compared to wild-type controls [79]. RNAi-mediated knockdown of EphB2, expressed by fibroblasts, prevented astrocyte aggregation at the injury border, increased neurofilament expression within the lesion, decreased fibronectin deposition, and promoted greater numbers of myelinated fibers [80]. However, it did not affect overall astrocyte reactivity or gross motor recovery [80]. Interestingly, studies of global deletion of EphA4 before dorsal hemisection SCI did not show changes in GFAP immunoreactivity [81,103], but did cause changes in border formation and fibronectin deposition 2 weeks post-injury [81].

Another approach has focused on immune cells, particularly macrophages, given their central role in driving fibroblast activation and recruitment. Hematogenous macrophage depletion reduces the density of Col1a1^+^ fibroblasts and impaired basal lamina formation between the astrocytic and fibrotic scar [58]. Diphtheria toxin receptor-mediated ablation of macrophages after complete crush SCI resulted in decreased collagen III and fibronectin deposition, lower spinal cord TGF-β1 levels, reduced pSMAD2 signaling, increased βIII-tubulin-positive nerve fibers, and improved functional recovery [100]. Similarly, conditional knockout of TGFB1 in macrophages reduced fibronectin deposition, increased nerve fiber density in the lesion area, lowered TGF-β1 levels, and decreased pSMAD2-positive cells, confirming the critical contribution of macrophage-derived TGF-β1 to fibrosis [100].

Direct genetic targeting of scar-forming cells has provided particularly informative results. Conditional knockout of TGF-β receptor 2 in type A pericytes reduced collagen III deposition, increased 5-HT and βIII-tubulin-positive nerve fibers, decreased pSMAD2-positive cells, and improved sensory and locomotor recovery, while increasing astrogliosis [100]. The importance of Ras signaling for cell division was leveraged using a “Rasless” system, in which Cre-recombination deletes all Ras genes, thereby preventing Ras-mediated proliferation [104]. When this approach was used to inhibit dividing type A pericytes after dorsal hemisection SCI, the effects on fibrotic scarring were dependent on the efficiency of recombination [40,105]. High-efficiency recombination and ablation led to severe tissue defects, complete absence of fibrotic scarring, and failure to close the injury site, consistent with previous findings [40,105]. Modest efficiency, however, reduced but did not eliminate fibrotic scarring, resulting in spontaneous regeneration of corticospinal and rubrospinal tract axons beyond the lesion and improved functional recovery [105]. Additional studies are required to determine the optimal level of fibrotic scar attenuation that balances scar reduction with preservation of tissue integrity. Studies using genetic mouse models to perturb the fibrotic scar after CNS injury are summarized in Table 3.

## 7. Comparative Insights from Other SCI Models

### 7.1. Neonatal Mice

Age-dependent effects on CNS repair are well-established, with axon regeneration and functional recovery declining sharply with increasing age after SCI [107]. In contrast, neonatal mice exhibit robust regenerative capacity and often achieve near-complete functional recovery without persistent scarring [108,109]. This marked difference from adult SCI, where both fibrotic and astrocytic scars form major barriers to repair, has made neonatal models a powerful tool for investigating scar-free healing and the role of fibrotic scarring in limiting regeneration.

Complete crush SCI at different postnatal ages reveals a clear age-dependent gradient in scarring and regenerative outcomes [108]. In adult mice, the injury site develops a stereotypical fibrotic scar densely populated with collagen I, fibronectin, and immune cells, while largely devoid of astrocytes, microglia, blood vessels, and axons [108]. In contrast, P2-injured neonates show a near-complete absence of collagen I/III and fibronectin, minimal PDGFRβ and immune cell presence, and repopulation of axons, astrocytes, microglia, and blood vessels, closely resembling uninjured tissue [100,108,110]. P2-injured mice also lack detectable active TGF-β signaling, effectively preventing the initiation of fibrotic scar formation [100]. For SCI at P12, however, pSMAD2 and PDGFRβ expression emerge, accompanied by small but detectable fibrotic scars, highlighting a transitional phase in which even “neonatal” mice begin to develop early scarring features and indicating a narrow developmental window for scar-free healing [100,108]. Interestingly, experimental depletion or functional perturbation of microglia in P2-injured mice disrupts regenerative bridge formation, stalls axon regrowth, and induces scar-like changes, suggesting a critical role of microglia in scar-free healing [108]. Further studies are needed to identify molecular signals by which microglia prevent fibrotic scar formation in neonatal mice and determine whether these mechanisms can be recapitulated in adult SCI.

### 7.2. Spiny Mouse

Acomys cahirinus, the spiny mouse, is a mammalian model of adult regeneration, capable of minimal scarring repair across multiple tissues, including skin, kidney, heart, skeletal muscle, and spinal cord [111,112,113,114,115,116,117,118,119,120,121,122]. Unlike typical adult mammals, in which injury triggers dense fibrotic scarring that impedes recovery, Acomys frequently regenerates functional tissue with markedly reduced fibrosis [111,116]. This attenuated fibrotic response makes the spiny mouse a valuable model for understanding how minimizing fibrotic scarring can promote neural repair after SCI.

In full-thickness skin biopsy punch wounds, fibroblasts invade the injury site in both Mus musculus and Acomys, but Acomys produces significantly less collagen [116]. Shotgun proteomic studies further reveal divergent collagen profiles, with Acomys exhibiting reduced collagen deposition despite upregulation in collagen remodeling [114]. In ear pinnae punch models, Mus maintains myofibroblasts in fibrotic tissue through 4 weeks post-injury (wpi), whereas Acomys displays only transient myofibroblast presence that resolves by 3 wpi, coinciding with complete wound closure and revascularization [113]. In chronic kidney injury, Mus develops increased collagen and extensive interstitial fibrosis, while Acomys shows no significant change in collagen content and near-complete absence of interstitial matrix fibrosis at 21 days post-obstruction [112].

In the context of SCI, Acomys exhibits reduced fibrotic scarring and enhanced regeneration. For example, after thoracic complete transection, Acomys achieves motor and bladder functional recovery by 8 wpi, including the ability to recover after a second SCI [111]. This functional improvement correlates with increased βIII-tubulin^+^ axons that penetrate and span newly formed bridging tissue [111]. Decreased collagen, injury site area, GFAP expression, and NGAL/LCN2 expression suggest attenuated astrocytic and fibrotic responses compared to Mus [111,115]. However, the lack of reliable antibodies and transgenic lines in Acomys prevents further detailed fibrotic scar characterization. Once these tools become available, future work can understand the differences in the activity, recruitment, and contribution of fibrotic-scar-forming cells between Acomys and Mus after SCI.

### 7.3. Zebrafish

Many organisms are capable of robust regeneration after CNS injury, including salamanders, axolotls, C. elegans, and zebrafish [123,124,125,126,127]. Zebrafish’s extensive capacity for scar-free neural repair after SCI, alongside their adequate system complexity and genetic and imaging tools, provides a particularly powerful vertebrate model.

As early as 1 dpi in larval zebrafish, when axons begin regenerating, PDGFRβ^+^ cells detach from blood vessels and migrate into the injury site, mirroring the behavior of fibroblasts and type A pericytes in mouse SCI [13,40,128]. Adult zebrafish transgenic lines labeling GFAP^+^ radial glia-like cells show proliferation around the central canal within 3 dpi, with cells accumulating at the lesion borders by 5 dpi [129]. By 3 wpi, glial bridges span the lesion, filled with proliferative cells that enable axon crossing [129]. This dynamic bridging contrasts sharply with mammalian astrocytes, which proliferate and migrate to the lesion edges but form a static, stagnant border around the fibrotic core rather than functional bridges. Nevertheless, in both systems, regenerating axons closely associate with GFAP^+^ glial processes, suggesting a conserved supportive role for glia in guiding axonal growth.

Beyond glial bridging, differences in fibrotic and ECM responses create a highly permissive lesion environment in zebrafish. While some axons follow glial processes, many regenerating axons navigate non-neural areas enriched with ECM and PDGFRβ^+^ cells, indicating broad remodeling into a growth-permissive milieu that is largely absent in mammals [130]. A central mechanism underlying this permissiveness is a switch in PDGFRβ^+^ cell-derived ECM composition. For example, after zebrafish SCI, there is an upregulation of pro-growth ECM genes, such as col12a1a/b, colocalizing with PDGFRβ^+^ cells [128,130]. PDGFRβ^+^ cells upregulate pro-growth components (cthrc1a, col12a1a/b, tenascin-C) while downregulating inhibitory molecules (lumican, mfap2, periostin, and collagen IV) [128,130]. This switch prevents inhibitory scarring, in contrast to mammalian fibrotic scars, which are dominated by persistent inhibitory ECM.

The concept of balanced permissive versus inhibitory ECM has also been studied in non-regenerative adult mammalian models. Traditionally, chondroitin sulfate proteoglycans, myelin-associated inhibitors, semaphorins, and certain collagens have been classified as inhibitory [43,82,131,132,133,134,135,136,137,138,139,140,141,142], while laminin and fibronectin have been considered growth-permissive [84,143,144]. However, recent perturbation and sequencing experiments suggest that these binary classifications may be overly simplistic, as axons often grow along mixed substrates when permissive cues sufficiently outweigh inhibitory signals. Whether the pro-growth ECM switches observed in zebrafish can be recapitulated in the adult mammalian spinal cord remains unknown.

### 7.4. Rat

Rats have long served as a highly translatable model for SCI research, more closely recapitulating key features of human SCI pathology than mice. While both rodent species share similarities in injury responses, there are notable differences in lesion architecture, inflammatory dynamics, and fibrotic scar organization. Understanding these distinctions is essential for accurate interpretation of mouse genetic studies and their validation in models with greater clinical relevance.

Following contusive SCI in rats, lesion-site cavitation begins to emerge around 2 dpi, a process absent in mice [145]. Along with tissue cavitation, rats develop larger lesions and a more severe breakdown of the blood–spinal cord barrier compared to mice [146]. While in both species the injury core is absent of GFAP^+^ astrocytes, in rats the central cavity is also devoid of ECM proteins and cells in general [84,146]. In contrast, the mouse injury core is densely filled with a distinct immune and fibrotic profile, including fibronectin-rich ECM [84]. Regardless, in both models, an astrocytic scar and fibrotic scar form along the injury site after SCI [84,145]. At 56 dpi in rat contusive SCI, Iba-1, fibronectin, and PDGFRβ localize prominently along the peripheral rim of the cavities, whereas all are present within the injury core in mice SCI [84,146]. Interestingly, rats exhibit greater overlap between GFAP^+^ astrocytic regions and fibronectin^+^/PDGFRβ^+^ fibrotic regions, suggesting that the fibrotic/glial scar is more intertwined in rats [84].

### 7.5. Non-Human Primates & Humans

Non-human primates and human post-mortem tissue provide high translational insight into fibrotic scar formation after SCI, serving as a bridge between rodent mechanistic studies and human pathology. In human post-mortem spinal cord samples from patients with compression or contusion/cyst-type SCI, regions of scar tissue are enriched with PDGFRβ^+^ cells and frequently bordered by reactive glia [53]. This fibrotic–glia accumulation and separation, stereotypical after SCI, was also observed in chronic multiple sclerosis spinal cord tissue [53]. In assessing post-mortem injured spinal cord tissue, TGF-β1 and TGF-β2 upregulation is detectable in macrophages, microglia, and astrocytes, suggesting TGF-β signaling is implicated in human SCI lesion site dynamics, such as in mouse SCI [147].

In a rhesus macaque transection SCI model, scRNA-seq identified heterogeneous fibroblast and pericyte populations expressing markers such as Dcn, Col1a1, Rgs5, and Kcnj8 [16]. Additionally, changes in SCI ECM expression patterns were also observed, in which Tnc and Cthrc1 initially increase and then decrease, FN1 expression rises while laminin subunits decrease, Col12a1 expression remains persistently reduced, and Postn and Mfap2 stay elevated until approximately 30 dpi [16]. This suggests that pericyte and fibroblast-driven scarring, as well as dynamic ECM regulation, are conserved across species after SCI.

## 8. Comparative Insights from CNS Non-SCI Models

Similarly, other CNS injury and disease models, including traumatic brain injury, stroke, and multiple sclerosis, exhibit comparable injury responses to SCI, characterized by both astrocytic gliosis and fibrotic scarring [48,53,69,148]. In these models, fibrotic scarring supports acute wound healing but may impede neural repair and recovery as well [3,48,53,69,149,150]. Therefore, it is equally important to elucidate the underlying mechanisms, identify the key cell types involved, determine optimal intervention timing, and develop effective strategies to modulate fibrotic scarring to promote neural repair and recovery. Understanding how fibrotic scarring diverges and is conserved across other CNS injury and disease models will provide valuable new insights into fibrotic scarring after SCI.

### 8.1. Traumatic Brain Injury

Traumatic brain injury (TBI) is a form of brain injury initiated by a mechanical insult that triggers immediate tissue damage, followed by cascades of neuroinflammation, edema, hypoxia, neurodegeneration, and scar formation. Fibroblasts and pericytes have both been implicated in fibrotic scarring after TBI. Lineage tracing of fibroblasts using Col1a2-CreER; Rosa26tdT in a controlled cortical impact model of TBI showed fibroblasts expanded into damaged regions, produced ECM components, and formed a lesion distinct from but adjacent to astrocytic gliosis by 14 dpi [69]. Similarly, GLAST^+^ lineage tracing after cortical stab wound TBI shows type A pericyte presence by 5dpi [53]. Inhibition of these type A pericytes results in decreased fibronectin and collagen 1 at the injury site at 14 dpi, alongside decreases in lesion core volume and PDGFRβ^+^ cells [53].

### 8.2. Multiple Sclerosis

Multiple sclerosis (MS) is a chronic neuroinflammatory disease characterized by immune cell infiltration, demyelination, and CNS white matter lesion formation [151,152]. Emerging evidence highlights prominent fibrotic scarring in MS lesions that contributes to a non-permissive environment, inhibiting remyelination and repair [48,153,154]. Human MS tissue from active white matter lesions shows a marked presence of ECM components, including fibrillar collagens, basement membrane collagen, and laminins, which collectively form a dense perivascular fibrotic scar [153,154]. In an EAE mouse model of MS, ECM deposition, PDGFRβ, and Col1a1-GFP cells rapidly localize to parenchymal lesions following initial immune cell influx and onset of motor symptoms, peaking around 10 days post-symptom onset (d PSO) and persisting chronically [48,54,155]. These Col1a1^+^ cells co-express PDGFRα and PDGFRβ [48,155], confirming a fibroblast-like identity similar to that observed in SCI [13]. Like perivascular fibroblasts and type A pericytes in SCI, these Col1a1^+^ cells detach and scatter from vessels during lesion formation [13,40,48]. Similarly, Col1a2 lineage tracing revealed a dramatic ~70-fold increase in traced cells within EAE MS lesions [48]. Ablation of Col1a2^+^ cells via herpes simplex virus thymidine kinase improved motor function and decreased ECM deposition [48]. Interestingly, cells labeled by Col1a2CreER^T2^ reporter mice had significant overlap with Col1a1-GFP expression (>80% colocalization in Col1a2CreER^T2^; tdTomato^fl/fl^; Col1a1-GFP mice), indicating that the majority of fibrotic scar fibroblasts are derived from cells already expressing collagen I before EAE induction. Whether a comparable pre-existing Col1^+^ population contributes to the fibrotic scar after SCI remains to be definitively tested, for example, by combining Col1a2CreERT2 with Col1a1-GFP reporters.

Gene expression analyses in PLP-induced relapsing–remitting EAE show early upregulation of MMP9, PDGFRβ, αSMA, collagen IV, fibronectin, and LOXL3, followed by late-phase increases in MMP9, NG2, PDGFRβ, CD31, collagen I, and LOXL3 [54]. When comparing late to early EAE, αSMA, collagen IV, and fibronectin expression are more prominent in the early phase, indicating phased ECM remodeling [54]. Importantly, fibrotic scarring in EAE is driven primarily by immune cell infiltration rather than demyelination alone. Inhibiting inflammation (with fingolimod) or using immune-free demyelination models (cuprizone) fails to produce fibrotic scars [48].

ScRNA-seq of Col1a1^+^ cells at 7 d PSO in EAE shows elevated expression of activated fibroblast genes [48]. Bulk and scRNA-seq analyses further implicate interferon-gamma (IFN-γ) signaling from T cells as a key driver of fibrotic scarring [48]. Conditional knockout of the IFN-γ receptor 1 specifically in Col1a2^+^ cells reduces scar formation (although less effectively than fibroblast ablation) without significantly affecting motor dysfunction, lesion size, or myelination [48]. In contrast, astrocyte-specific overexpression of IFN-γ does not induce spontaneous scarring, underscoring that immune-fibroblast crosstalk, rather than astrocyte-derived signals alone, is central to fibrosis in MS [48].

### 8.3. Stroke

Stroke is a brain injury due to disrupted blood flow, resulting in immediate neuronal death, inflammation, edema, and scar formation [149,156]. Human brain sections from stroke patients reveal prominent collagen fiber deposition within active white matter lesions [153]. In the endothelin-1 ischemic stroke mouse model, Col1a1 accumulates in white matter lesions and persists in the brain parenchyma during the chronic phase [153]. Col1a1-positive signals also colocalize with desmin-positive pericytes, vascular smooth muscle cells, and CCR2^+^ macrophages in ischemic lesions at 7 dpi [153]. Electron microscopy demonstrates collagen fibers surrounding blood vessels at both 7 and 21 dpi, supporting the idea that perivascular cells actively secrete collagen within white matter lesions [153].

Col1a2^+^ lineage-tracing after PT stroke shows fibroblasts expand into damaged regions, produce ECM components, and form a distinct fibrotic lesion adjacent to parenchymal astrocytic gliosis by 14 dpi [69]. Fibroblasts first localize near the wound border by 4 dpi, expand substantially by 7 dpi, infiltrate the contracting lesion by 14–21 dpi, and persist even at 1-year post-injury [69]. Lineage tracing of ATP13a5^+^, NG2^+^, and Acta2^+^ cells shows minimal contribution from pericyte or vascular smooth muscle cell lineages to stroke injury sites, mimicking pericyte-specific lineage tracing after SCI [69]. These findings collectively suggest that fibroblasts, rather than pericytes, are the predominant source of fibrotic scarring in stroke. However, GLAST^+^ lineage tracing of type A pericytes shows labeled cells in the injury site after ischemic stroke, suggesting different stroke models recruit different cells or recombined GLAST^+^ cells are fibroblasts [53]. Dual-reporter analysis with Col1a1GFP; Col1a2creER; Rosa26tdTomato revealed that after stroke, approximately 50% of GFP^+^ cells were also tdTomato^+^, with delayed lineage tracing showing near 100% overlap [69]. Critically, future lineage-tracing experiments must carefully consider tamoxifen timing, as the genetic profile of scar-forming cells and promoter expression likely changes dynamically in response to injury, potentially altering the targeted cell populations over time. Fibroblast and pericyte genetic labeling studies in non-SCI CNS models are summarized in Table 4.

Myeloid and glial cells, similar to SCI, play a role in fibrotic scar formation after stroke. Depleting infiltrating myeloid cells through clodronate liposome treatment after PT stroke reduced scar fibroblasts, ECM, and increased lesion size [69]. More specifically, TGFβ1 conditional knockout from Cx3cr1^+^ cells decreased injury site ECM, suggesting that myeloid-derived TGFβ1 contributes to the injury-induced fibroblast response [69]. This is complemented by the findings that deletion of TGFBR2 in Col1a2^+^ fibroblasts after PT stroke or transient middle cerebral artery occlusion reduced lesional fibroblasts and associated ECM but increased lesion size, sub-acute mortality, and neuronal degeneration despite intact glial scarring [69]. The αvβ8-blocking antibody ADWA11, which blocks αv-paired integrin-mediated TGFβ cytokine activation, also reduced injury site fibroblasts and ECM deposition, but not lesion areas [61]. Itgb8 was shown to label perilesional astrocytes, and astrocyte-specific knockout of Itgb8 had a similar phenotype to ADWA11 treatment, suggesting that αvβ8 integrin on lesion-adjacent glial cells licenses TGF-β-mediated myofibroblast expansion [69]. Pharmacological inhibition of PDGF signaling, to prevent myofibroblast generation, reduced fibrotic scarring after ischemic stroke [150], while genetic deletion of myofibroblasts modestly increased brain lesion size and reduced lesional ECM in stroke models [69].

## 9. Conclusions

This review synthesizes the current understanding of fibrotic scarring after SCI. Fibrotic scar-forming cells, primarily fibroblasts with contributions from a subset of GLAST^+^ type A pericytes, rapidly activate, proliferate, and migrate into the lesion core within the first week post-injury [13,16,18,40,53,105]. These cells deposit key ECM proteins and establish a persistent fibrotic scar that is sharply separated from the surrounding astrocytic scar [13,16,141]. This process is driven by multiple signaling pathways, such as TGF-β, PDGF, CXCL4, Eph/ephrin-, and Plexin [63,74,75,76,77,78,79,80,81,101,102]. Genetic and pharmacological interventions targeting the fibrotic scar, including inhibition of TGF-β, PDGF, periostin, or CXCL4, as well as selective ablation or attenuation of fibroblast/pericyte populations, consistently reduce ECM deposition, decrease scar density or size, enhance axonal regrowth across the lesion, and improve locomotor and sensory recovery [75,81,100,102,105]. Beyond matrix deposition, however, fibroblasts and other mesenchymal cells likely influence SCI outcomes through secretion of cytokines, chemokines, and growth factors whose roles in tissue repair, immune regulation, and neuronal survival remain poorly understood [1,2,19]. This has important therapeutic implications, as interventions designed to suppress fibrotic scarring may simultaneously remove beneficial reparative signals, underscoring the need to determine not only which fibrotic mechanisms inhibit regeneration, but also which aspects of the fibroblast response should be preserved and when interventions should be applied [40,69,105].

Comparative analyses across models highlight both conserved mechanisms and important species- and injury-specific differences. Neonatal mice, spiny mice, and zebrafish demonstrate that robust functional recovery is possible with minimal or no fibrotic scarring, driven by absent or transient myofibroblast activity, pro-regenerative ECM remodeling, and supportive glial responses [108,111,130,157]. Non-human primate and human post-mortem data support broad conservation of fibroblast-driven scarring and TGF-β signaling [16,53,147,158]. Insights from non-SCI CNS models (TBI, stroke, and multiple sclerosis) further reinforce the central role of fibroblasts and immune-fibroblast crosstalk, while revealing context-dependent differences in pericyte contributions [48,53,69].

These advances have been enabled by powerful tools, including Cre-lox lineage tracing, single-cell and spatial transcriptomics, and conditional genetic manipulations. Collectively, the evidence establishes the fibrotic scar as a central, modifiable determinant of the lesion microenvironment that influences neuroprotection, neuroregeneration, and neuromodulation. Nevertheless, important gaps remain (summarized in Table 5), including resolving ambiguities in fibroblast/pericyte markers, defining injury- and model-specific fibroblast heterogeneity, elucidating signals that sustain the astrocytic–fibrotic border, and identifying the optimal degree and timing of scar modulation. Addressing these questions will facilitate the development of nuanced therapies that balance the beneficial and inhibitory functions of fibrosis. Such progress holds promise not only for SCI but also for other fibrotic CNS conditions, including TBI, stroke, and multiple sclerosis.

## Figures and Tables

**Table 1:** Markers labeling fibroblasts, pericytes, or both.

Fibroblasts	Pericytes	Fibroblasts & Pericytes
Non-fibrillar collagens [15,17,18,35]	CSPG4 [17,18,24,25,37,45,46,47,48]	PDGFRβ [17,37,42,43,44,45,47]
Fibrillar collagens [15,17,18,35,48]	Abcc9 [17,18,32,34,37,48]	Anpep [15,17,18,29,37]
PDGFRα [15,16,17,18,20,21,49]	Desmin [26,27]	Hic1 [49]
Decorin [17,18,48,49]	Rgs5 [15,17,18,30,31,37,49,50]	Slc1a3 [17,18,40,41]
Lama1 [17]	Dlk1 [32]	
Lumican [15,16,17,18,35,49]	Cd248 [17,33]	
Pi16 [15,18]	Atp13a5 [15,18,34,35]	
Periostin [18]	Tbx18 [15,36,51,52]	
Fibronectin 1 [18]	Vtn [17,18,35,37]	

**Table 2:** Prior fibroblast & pericyte SCI genetic labeling studies.

Promoter	SCI Model	Present in Injury Site	Refs.
Col1a2	Complete transection	✓	[16]
	Complete crush	✓	[16]
GLAST	Complete crush	✓	[18,53]
	Dorsal funiculus/dorsal hemisection	✓	[40,53]
	Contusion	✓	[18]
Col1a1	Complete crush	✓	[18]
	Contusion	✓	[13,18,58]
	Dorsal hemisection	✓	[13]
PDGFRβ	Complete crush	✓	[16,18]
	Contusion	✓	[18]
	Complete transection	✓	[16]
NG2	Contusion	✕	[13]
	Complete transection	✕	[16]
Myh11	Complete transection	✕	[16]
	Complete crush	✕	[16]
Crabp2	Complete transection	✓	[16]
	Complete crush	✓	[16]

**Table 3:** Prior fibroblast & pericyte CNS genetic manipulation studies.

Promoter	Target	Model	Finding
GLAST	TGFBR2	Complete Crush SCI	Reduced ECM & lesion size, increased axon regrowth [100]
	Rasless	Dorsal funiculus/Dorsal hemisection SCI	Decreased PDGFRβ, tissue defects with increasing recombination efficacy [40,105], increased axon regrowth [105]
		TBI	Decreased ECM, PDGFRβ, and lesion core volume [53]
Col1a2	TGFBR2	Stroke	Decreased ECM, increased lesion size [69]
	Cxcl12	Stroke	No change in lesion area or ECM [69]
	Ifngr1	Multiple sclerosis	Reduced ECM, no change in lesion area [48]
	HTK	Multiple sclerosis	Reduced ECM, no change in lesion area [48]
Col1a1	Rasless	Complete Crush SCI	Reduced PDGFRβ [18]
Cthrc1	DTA	Stroke	Decreased ECM, increased lesion size [69]
RGS5	KO	Stroke	Decreased PDGFRβ, no change in lesion area or ECM [106]

**Table 4:** Prior fibroblast & pericyte labeling in non-SCI CNS models.

Promoter	Injury Model	Present in Injury Site	Ref.
Col1a2	Stroke	✓	[69]
	Traumatic brain injury	✓	[69]
	Multiple sclerosis	✓	[48]
GLAST	Traumatic brain injury	✓	[53]
	Multiple sclerosis	✓	[53]
	Stroke	✓	[53]
	Glioma	✓	[53]
Col1a1	Multiple sclerosis	✓	[48]
	Stroke	✓	[69]
	Traumatic brain injury	✓	[51]
NG2	Stroke	✕	[69]
	Multiple sclerosis	✕	[48]
ATP13a5	Stroke	✕	[69]
aSMA	Stroke	✕	[69]
	Multiple sclerosis	✕	[48]
Gli1	Stroke	✓	[69]
Twist	Stroke	✓	[69]
Acta2	Stroke	✕	[69]
Cthrc1	Stroke	✓	[69]
Tbx18	Traumatic brain injury	✕	[51]
Hic1	Stroke	✓	[49]
PDGFRα	Stroke	✓	[49]

**Table 5:** Gaps in the field.

Category	Questions
Profiling scar forming cells	What are distinguishing markers for perivascular and meningeal fibroblasts?
	2. How does the genetic profile of scar forming cells change after SCI?
	3. How does the composition of fibrotic scar cells vary with injury model, severity, or level?
Fibrotic scar manipulation	Does PDGF signaling play a role in SCI fibrotic scar formation? If so, can this synergize with TGF-β signaling?
	2. How does manipulating the fibrotic scar impact the injury site microenvironment and axon regeneration?
	3. What is the optimal degree of scar attenuation?
Fibrotic scar crosstalk	What are the mechanisms by which the fibrotic and astrocytic scar work together to establish the injury border? What is the optimal timing for therapeutic modulation of fibrotic scarring to preserve beneficial neural repair functions while reducing chronic inhibitory effects?

## Data Availability

No new data were created or analyzed in this study.
